# Asymptomatic Intestinal Parasitic Infestations among Children Under Five Years in Selected Communities in the Ho Municipality, Ghana

**DOI:** 10.4314/ejhs.v30i6.3

**Published:** 2020-11

**Authors:** GE Kpene, SY Lokpo, JG Deku, E Agboli, PK Owiafe

**Affiliations:** 1 Department of Medical Laboratory Sciences, School of Allied Health Sciences, University of Health and Allied Sciences, Ho, Ghana; 2 Department of Epidemiology and Biostatistics, School of Public Health, University of Health and Allied Sciences, Hohoe, Ghana

**Keywords:** Pathogens, infestations, parasites, children under 5 years

## Abstract

**Background:**

The study investigated intestinal parasitic infestations (IPIs) and possible risk factors associated with asymptomatic children under five (5) years in five (5) selected communities in the Ho Municipality.

**Methods:**

The study design was cross- sectional, with a simple random sampling technique involving 150 asymptomatic children under 5 years from 5 selected communities (Klave, Hoe, Freetown, Dave and Godokpe) in the Ho Municipality. A questionnaire was used to obtain socio-demographics and other relevant parameters. Direct wet preparation, formol-ether concentration and Modified ZN staining techniques were used for the identification of intestinal parasites from participants' stool samples. The Fisher's exact test and binary logistic regression analysis were used to determine the difference in IPIs proportions and assess the risk factors associated with IPIs respectively.

**Results:**

The overall IPIs cases was 14% (21/150). Cryptosporidium spp was most predominant [5.3% (8/150)], followed by Entamoeba spp [3.3% (5/150)], Cyclospora cayetenensis [2.7% (4/150)], Ascaris lumbricoides [1.3% (2/150)], Giardia lamblia [0.7% (1/150)] and Strongyloides stercoralis [0.7% (1/150)]. Children in rural communities (23.4%) recorded significantly higher case rate compared to those in urban communities (9.8%0), (p=0.04). Lower educational attainment of mother [OR=0.55, 95% CI (0.37 – 0.83), p-value = 0.015] and residence in rural communities [OR = 0.53, 95% CI (0.33–0.88)], p-value = 0.025] were significantly associated with IPIs.

**Conclusion:**

Asymptomatic IPIs are quite prevalent among children under 5 years in the Ho Municipality. The study thus recommends active sensitization programs for parents/guardians on preventive measures and school health programs should be instituted in rural communities.

## Introduction

Several asymptomatic intestinal parasitic infestations (IPIs) go undetected especially among children and are mostly untreated (1). Among the major clinical manifestations of IPIs, diarrhea with abdominal cramping, vomiting, flatulence and weight loss rank as the common symptoms with the undernourished and immunocompromised patients usually having severe symptoms (2). Approximately, about half of the world's population, mainly inhabitants of the tropics and subtropics, are infected with parasitic intestinal helminth (3). It is estimated that *Ascaris lumbricoides*, hookworm and *Trichuris trichiura* infestations, coupled with schistosomiasis are responsible for more than 40% of the global morbidity from all tropical infestations (3). Other intestinal parasites include: *Cryptosporadium parvum, Entamoeba histolytica*, and *Gardia lamblia. Cryptosporidium* is a human intestinal coccidian known to cause diarrhea among immunocompromised individuals including malnourished children and people living with HIV/AIDS. The infective stage (oocyst) is transmitted via faecal-oral route and its occurrence is shown to be affected by climate, high in the rainy season but low during the dry season (4,5). *E. histolytica* is a human intestinal pathogen that affects more than 50 million people worldwide, resulting in 100,000 deaths per annum (6). It is transmitted by the ingestion of infective cyst in contaminated food and water. Unlike *C. parvum* that is climate dependent, the distribution of *E. histolytica* is related more to inadequate environmental sanitation and poor personal hygiene. Diarrhea caused by *E. histolytica* is the third leading cause of death (7). Risk factors such as place of residence, age, ingestion of raw vegetables, and drinking water quality were significantly associated with diarrhea caused by *E. histolytica* (7). *G. lamblia*, another enteroparasite, is transmitted by the faecal-oral route. Its trophozoite is pear shaped with one or two transverse, claw-shaped median bodies and has a direct life cycle (8). In developing countries such as Kabul Afghanistan, Israeli Bedouin and South Africa, there is a high incidence, and data suggest that chronic giardiasis can cause long-term growth retardation (9–11). IPIs can increase susceptibility to other gastrointestinal pathogens and thereby leading to complications in the individual (12,13).

In Ghana, IPI ranks among the top five morbidities (14). Afranie and colleagues in a retrospective study recorded IPIs rate of 10% among patients who visited the Ho Teaching Hospital from 2012 to 2016 (15). This hospital-based data may be skewed, thus cannot be said to be representative of the entire population of the Ho Municipality. Moreover, a previous study conducted in the Volta Region has identified gaps relating to the diagnostic performance of clinical laboratories where none of the laboratories sampled attained the WHO recommended rating (16). Hence, with about 9% of the population being children <5 years in the Ho Municipality, better understanding of a community-based IPI rate and associated risk factors would serve to be a valuable contribution to public health interventions in the Municipality. In this study, we aimed to investigate the IPIs and possible risk factors associated with asymptomatic children under 5 years in five communities in the Ho Municipality.

## Patients and Methods

**Study area**: The Ho Municipality is one of the five (5) municipalities within the Volta Region. It is made up of 21 urban communities and 15 rural communities. Ho Municipal is the capital town of Volta Region which was established under the Legislative Instrument (LI) 2074 of 2012. The Municipality lies between latitude 6°20″N and 6°55″N and longitude 0°12′E and 0°53′E, sharing boundaries with Adaklu and Agotime Ziope Districts to the South, Ho West District to the North and West, and the Republic of Togo to the East. The land area of the Municipality is 2,361 square kilometers forming 11.5% of the total land area of the Volta Region (17). The population of the Municipality in 2010 was 177,281 (83,819 males and 93,469 females) of which 19,618 were children under 5 years. A total of 110,048 of the population, representing 62.1% were living in urban areas compared to the 37.9% (67,233 persons) in rural areas (17). In the Municipality, children under 5 years recorded a significant number of diarrhea cases between 2012 and 2016 (18).

**Study Site description**: Two rural (Klave and Hoe) and three urban communities (Freetown, Dave and Godokpe) were conveniently selected in the Ho Municipality. Klave and Hoe are small farming communities with a Health Centre at Shia, a nearby community serving their health needs. Freetown, Dave and Godokpe are also small communities in the Ho Municipality, and the people of these communities mostly seek healthcare at the Ho Polyclinic.

**Study design and study population**: The study design was cross-sectional where we employed a simple random sampling technique. A structured questionnaire was used to collect data from parents/guardians of study participants. These included general demographic characteristics and socio- economic lifestyles. The study population included children under 5 years in the selected communities in the Ho Municipality.

**Sample size determination**: Using the Raosoft sample size calculator (http://www.raosoft.com/samplesize.html) with 5% margin of error, 95% confidence interval, 245 population size, and 50% response distribution, a minimum number of 150 respondents were recruited into the study.

**Sample collection**: Parents/guardians of each child were educated and asked to collect about 5g of fresh stool sample into a clean leak-proof container. These containers had tight-fitting leak-proof lids, without disinfectant or detergent residue. Each stool sample collected was transported in an icebox to the laboratories at the Shia Health Center and University of Health and Allied Sciences for investigations within a maximum of 2 hours after collection.

**Laboratory Investigation**

**Sample preparation**: Each sample collected was processed using the direct wet preparation, formol ether concentration technique and modified ZN staining technique previously described by Cheesbrough (8). The samples were centrifuged using a Benchtop Centrifuge [Thermo Scientific™, UK]. The processed samples were subsequently examined microscopically using x100 (modified ZN staining technique) and x40 (direct wet preparation and formol ether concentration technique) objectives [Leica DM300, China].

**Direct wet mount**: One gram of the stool sample was emulsified with a drop of normal saline using an applicator. This was done on a clean grease free slide and covered with a cover glass.

**Formol ether concentration**: One gram of the stool sample was emulsified in 4 ml of formol saline. A further 4 ml of formol saline was added and mixed thoroughly by shaking. The resulting suspension was sieved into a beaker, transferred into a polypropylene centrifuge tube after which 4ml of ether was added. The tube was stoppered and shaken for 15 seconds to ensure a homogenous mixture. The stopper was loosened carefully with tissue wrapped around the top of the tube and centrifuged immediately at 3000rpm for 1 minute. After centrifugation, supernatant layers (ether, faecal debris and the formol saline) were discarded. A drop from the sediment resuspended was transferred onto a slide and covered with a cover glass.

For formol ether oocyst concentration technique, centrifugation was done at a low speed of 1000rpm for 1 minute. Using a Pasteur pipette, the entire column of fluid suspension below the faecal debris and ether layers was carefully aspirated and transferred into another centrifuge tube. Formol saline was added to make the volume up to 10–15 ml and centrifuged at 3000rpm for 10 minutes. Finally, the supernatant was removed, and the bottom of the tube was tapped to resuspend the sediment. The resuspension was transferred onto a clean grease free slide to prepare a smear for staining.

**Modified Ziehl-Neelsen (ZN) stain**: The modified Ziehl-Neelsen staining method was carried out after smears were prepared, air-dried and fixed in methanol for 2 minutes. The smears were primary stained (carbol fuschin) for 15 minutes, decolorized with 1% HCl for 10–15 seconds and counter stained with methylene blue for 30 seconds. The smears were finally washed with water and allowed to air dry.

**Statistical analysis**: Data were entered into the Microsoft Excel 2013 spreadsheet for cleaning and validation. All statistical analyses presented were performed using IBM Statistical Package for the Social Sciences version 22.00 [SPSS Inc, Chicago, USA (http://www.spss.com)]. Descriptive statistics were used to summarize the socio-demographic characteristics of the study participants. Fisher's exact test was used to determine the difference in the proportions of IPIs between the rural and urban communities, and binary logistic regression analysis was done to assess the risk factors associated with IPIs among the participating children. P-values <0.05 were considered significant.

**Ethics**: Ethical clearance was given by the Ethical Review Committee of the University of Health and Allied Sciences with Protocol ID Number: UHAS-SAHS-ERSC:012A/2017. Assent was given by participants' parents/guardians. Written permission was sought from the various schools and child welfare clinics visited for the study.

## Results

A total of 150 children aged between 4 and 59 months took part in the study. Out of this number, 15(10.0%) were residents in Klave, 43(28.7%) in Dave and 40(26.7%) in Freetown while 32(21.3%) were living in Hoe and 20(13.3%) in Godokpe. There were more males, 78(52%), compared to females 72(48%). At the time of this study, 88(58.7%) of the mothers had completed Senior High school, 125(83.3%) married while 97(64.7%) had informal occupation.

From Table 1, majority of participants [54(36%)], were between 48 and 59 months, followed by those between 36 and 47 months, and the least found among those less than 6 months.

**Table 1:** Distribution of age category and gender of study participants

Age Category (months)	Total	Female	Male	p-value
**<6**	3(2.0)	1(0.7)	2(1.3)	
**6 – 11**	10(6.7)	4(2.7)	6(4.0)	
**12 – 23**	18(12.0)	8(5.3)	10(6.7)	
**24 – 35**	23(15.3)	7(4.7)	16(10.7)	
**36 – 47**	42(28.0)	23(15.3)	19(12.7)	
**48 – 59**	54(36.0)	29(19.3)	25(16.7)	0.4254

Data are presented as figure and percentages in parentheses. P-value significant at <0.05

**Figure 1:**
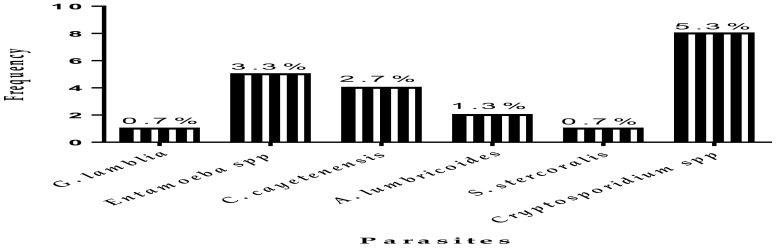
Distribution of intestinal parasites identified in the selected communities

Four (4) protozoan parasites (*Entamoeba spp, Cryptosporidium spp, G. lamblia* and *C. cayetenensis)* and two helminths (*S. Stercoralis* and *A. lumbricoides*) were identified from both formol ether concentration and direct wet preparations. IPI cases were 14% (21/150) with parasite specific cases as follows: *Cryptosporidium spp* 5.3% (8/150), *Entamoeba spp* 3.3% (5/150) and *C. cayetenensis* 2.7% (4/150), as well as *A. lumbricoides* 1.3% (2/150). However, *G. lamblia* and *S. stercoralis* recorded 0.7% (1/150) each (Figure 1).

There was a significant difference in the distribution of IPI cases between the study communities with the rural communities, [11(23.4%)], recording higher proportions compared to the urban communities [10(9.8%)], p=0.04. Additionally, *Entamoeba spp* infestation was the most recorded in the rural communities (8.5%) compared to *Cryptosporidium spp* in the urban communities (4.9%) (Table 2).

**Table 2:** Distribution of Intestinal Parasites among the selected communities

Parasite	Rural	Urban	p - value
**Present**	11(23.4)	10(9.8)	
**Absent**	36(76.6)	92(90.2)	**0.041**

***C. parvum***	3(6.4)	5(4.9)	
***E. histolytica***	4(8.5)	1(1.0)	
***C. cayetenensis***	3(6.4)	1(1.0)	
***A. lumbricoides***	0(0.0)	2(2.0)	
***S. stercoralis***	1(2.1)	0(0.0)	
***G. lamblia***	0(0.0)	1(1.0)	

Data are presented as frequency and percentages in parentheses. p–value significant at <0.05

Children of mothers with lower educational level (none and primary level of education) were observed to be 73% more likely to record IPI compared to those whose mothers had higher levels of education (secondary and tertiary) [OR=1.73; p-value=0.015]. Children who were resident in rural communities were 51% more likely to have IPIs compared to those who resided in urban communities [OR=1.51; p-value=0.025]. However, child's age, gender and washing bowls immediately after meals were not found to significantly account for IPI (Table 3).

**Table 3:** Binary logistic regression analysis of factors associated with intestinal parasitic infestations among study participants

Factor	n (%)	OR	95% CI	p-value
**Age of Child (Months)**				
<24	6(19.4)	0.68	0.316 – 1.454	
≥24	15(12.6)	1.13	0.850 – 1.499	0.335
**Gender of Child**				
Female	8(11.1)	1.30	0.735 – 2.308	
Male	13(16.7)	0.81	0.558 – 1.186	0.327
**Mother's Educational Status**				
Lower	13(22.8)	0.55	0.365 – 0.832	
Higher	8(8.6)	1.73	0.989 – 3.026	**0.015**
**Occupation of Mother**				
Unemployed	8(24.2)	0.51	0.266 – 0.973	
Employed	13(11.1)	1.30	0.921 – 1.841	0.055
**Washing Bowls**				
No	2(22.2)	0.57	0.127 – 2.559	
Yes	19(13.5)	1.05	0.904 – 1.208	0.463
**Location Type**				
Rural	11(23.4)	0.53	0.325 – 0.872	
Urban	10(9.7)	1.51	0.955 – 2.401	**0.025**

Data are presented as frequency and percentages in parentheses with p-values significant at <0.05. OR: Odd Ratio, CI: Confidence Interval

## Discussion

The IPIs cases found in this study (14%) were lower than those reported in other studies from Ethiopia (26.6%), Zambia (19.6%) and Pakistan (19.6%) (19–21). The intestinal parasites identified in this study included *G. lamblia, A. lumbricoides, Cryptosporidium spp* and *S. stercoralis, spp* as well as *C. cayetenensis.* However, co-infestation with *Trichuris* was not seen although this is a common observation (19). The most commonly identified parasite in this study was *Cryptosporidium spp* which is similar to the finding of a study conducted in Tanzania (22). In Zambia, *A. lumbricoides* was the most identified parasite (21), whereas *E. histolytica* was the predominant parasite in Ethiopia (20). The variations in the predominant parasite type observed accross the various geographical locations could possibly be due to the differences in the age, hygiene practices and parental socio-economic status as well as the different sources of infestations, climatic conditions and laboratory techniques employed in the identification of the parasites (15,23–25).

The only case of *G. lamblia* recorded in this study (0.7%) was proportionaly far lower than the 10.0% reported in the Odododiodio Constituency in the Greater Accra Region (26) and 89.5% at Agogo in the Asante Akim North Municipality of the Ashanti Region (27). Also, *E. histolytica* cases in this study were 3.3% which was lower than the 39.8% reported in the Bawku District of Northern Ghana (28). The lower rate of intestinal flagellate and *Entamoeba spp* infestation recorded in our study compared to those found in other parts of Ghana could be due to the difference in the population characteristics. While we recruited asymptomatic children aged between 4 and 59 months into our study, those conducted in Accra and Agogo comprised asymptomatic children aged 2–6 years and symptomatic children less than 18 years respectively. Moreover, the study in the Bawku District included 12 villages compared to the only five communities selected for this study. *A. lumbricoides* and *S. stercoralis* were the only helminths identified in our study, with a combined case rate of 2% (3/150). *Ascaris/Trichuris* transmission are generally observed to be higher in rural areas (19). However, in urban slums, their acquisition is probably related to poor sanitary conditions or contaminated water supplies (19).

*C. parvum* infestation was noted to be associated with rainfall (22). However, cryptosporidiosis is often asymptomatic and almost always self-limiting in immunocompetent hosts, but may be severe and life-threatening in patients with compromised immunity such as those with Acquired Immunodeficiency Syndrome (AIDS) or severe malnutrition. Eight (5.3%) children from the current study were infected with *Cryptosporidium spp*. Early childhood cryptosporidiosis has been associated with growth retardation, cognitive deficits and a higher overall risk of mortality (29). A possible reason for this observation could be due to mothers' unhygienic practices at home and improper care of the child. Moreover, a single dose of mebendazole which is given during school deworming programs is unlikely to treat infestations with *Cryptosporidium spp* (30).

In this study, we identified significant associations between residence in rural communities and lower educational status of mother with IPIs. Children who were living in rural communities were about twice as likely of recording IPIs compared to urban community dwellers. Water supplies in urban areas are relatively cleaner, and thus decrease the probability of drinking contaminated water. However, the activities of rural folks including farming, fetching water from streams and rivers as well as swimming brings, especially children, into close contact with soil and contaminated water. Therefore, these activities could increase the risk of ingestion and penetration of the infective stage of the parasites into the body (27). Hookworm and strongyloides are also known to be transmitted by penetration into the body. However, we identified only one case of strongyloides infestation in this study. The parents' educational status may influence the hygienic practices at home thus reducing the risk of infestation among children. According to Tyoalumun*et al.* (31), educated parents are likely to discipline their children to prevent them from playing in the sand and other areas that would likely increase their risk of infestation.

There are few limitations worth mentioning in this study. The sample size is relatively small; hence, a larger sample size should be used in future studies. However, findings from this study could provide a snapshot of the parasitic infestation situation in the study's jurisdiction. Also, other potential risk factors such as source of drinking water, toilet facilities used at home, number of children and net income of household were not evaluated in the study. Furthermore, our focus in this study was on maternal influence on childhood intestinal parasitic infestations; hence, paternal data was not obtained. Finally, due to the cross-sectional study design we employed, cause and effect relationships cannot be established.

In conclusion, asymptomatic intestinal parasitic infestations are quite prevalent among children under 5 years in the Ho Municipality. We therefore, recommend active sensitization programs for parents/guardians on preventive measures, and school health programs should be instituted in rural communities.
